# Supplementary material for: Influence of Sexual Dimorphism, Aging, and Differential Cell Capture Efficiency of Blood Separation Systems on the Quality of Platelet-Rich Plasma
Source: J Clin Med. 2022 Mar 18;11(6):1683. doi: 10.3390/jcm11061683 (PMC8953315; doi:10.3390/jcm11061683)
Supplement: Supplementary file 1 [file jcm-11-01683-s001.zip › jcm-1606300-supplementary.pdf]

Influence of Sexual Dimorphism, Aging, and Differential Cell Capture Efficiency of Blood Separation Systems on the Quality of Platelet-Rich Plasma

Figure S1. Blood cell count in WB and final products concentrated by the Easy kit system: (a) platelets; (b) WBCs (as a whole), neutrophils, lymphocytes, and monocytes.

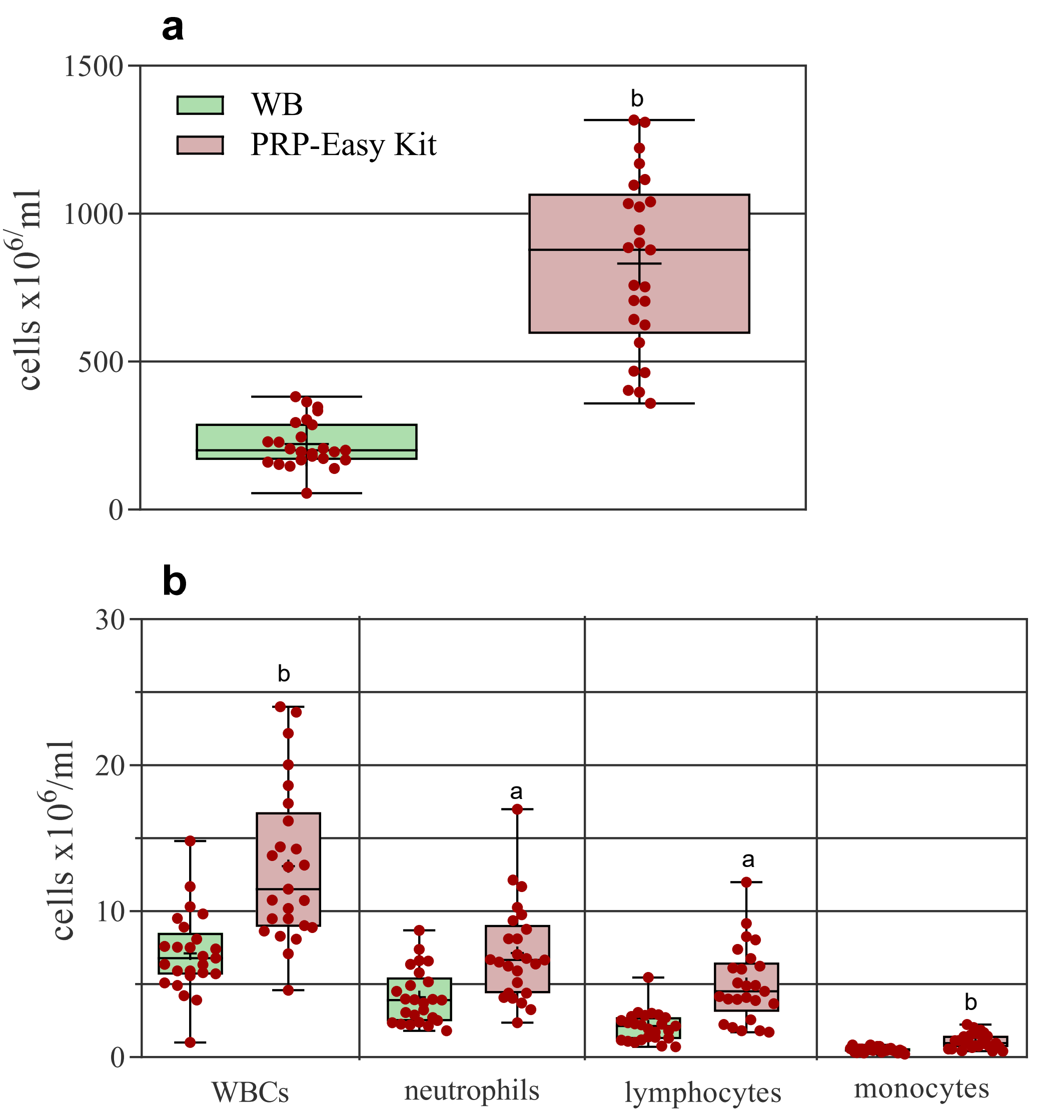

Median, line in the middle of the IQR box; mean, +; whiskers, minimum to maximum.
[a] P≤0.002 (U test). [b] P<0.001 (independent *t*-test) for WB vs. PRP products.

Table S1. Descriptive data of the sample population by sex.

	**Male Group n=13**		**Female Group n= 13**		***P-value***
	**Mean ± SD (range)**	**Median (IQR)**	**Mean ± SD (range)**	**Median (IQR)**	
Age (years)	62.09±24.30 (29.00-92.00)	64.00 [48.00]	62.50 ± 23.65 (31.00– 93.00)	70.00 [49.75]	0.826[a]
Weight (Kg)	75.36±17.29 (51.00-113.00)	76.00 [22.00]	69.21± 15.51 (52.00 – 103.00)	68.00 [23.75]	0.359[b]
Height (cm)	176.80±9.12 (166.00-193.00)	174.00 [14.00]	163.6± 6.23 (152.00- 174.00)	164.50 [10.30]	<0.001[b]
BMI (Kg/m2)	24.11±5.31 (17.75-34.11)	22.22 [8.00]	25.76± 5.05 (18.34- 34.58)	24.95 [7.66]	0.436[b]

Abbreviations: BMI, body mass index; SD: standard deviation; range (min–max); IQR: interquartile range; [a] Mann–Whitney U test; [b] independent *t*-test. A value of $p < 0.05$ (with a 95% confidence interval) was considered statistically significant.

Figure S2. PRP/WB ratio relationships with blood cell density ((a) platelets and WBCs; (b) neutrophils, lymphocytes, and monocytes) and (c) with CLR (neutrophils, lymphocytes, or monocytes/WBC ratio).

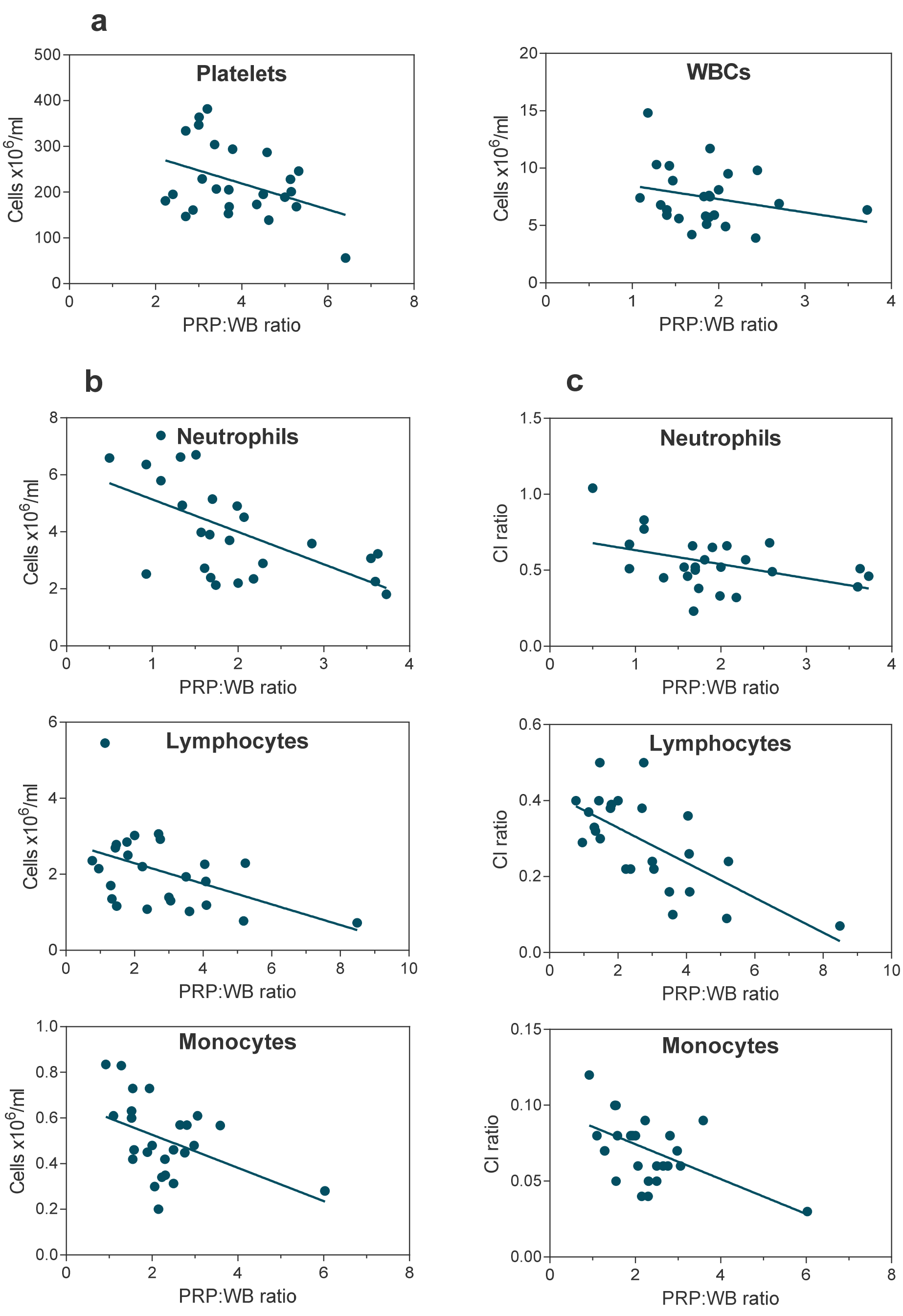

A: platelets, y=333.00 -28.50x; WBCs, y=9.62-1.16x.
B: neutrophils, y=6.27-1.14x; lymphocytes, y=2.83-0.27x; monocytes, y=0.67-0.07x.
C: neutrophils, y=0.72-0.09x; lymphocytes, y=0.42-0.05x; monocytes, y=0.09-0.01x.
According to the linear equation y=$y$intercept + slope*x, where y is the density or the CL ratio, intercept is the Y value where the line intersects the Y axis, slope is the slope of the line, and x is the PRP/WB ratio.
